# An approach to evaluate the topological significance of motifs and other patterns in regulatory networks

**DOI:** 10.1186/1752-0509-3-53

**Published:** 2009-05-19

**Authors:** Björn Goemann, Edgar Wingender, Anatolij P Potapov

**Affiliations:** 1 Department of Bioinformatics, Medical School, Georg August University of Göttingen, Goldschmidtstrasse 1, D-37077 Göttingen, Germany; 2 BIOBASE GmbH, Halchtersche Strasse 33, D-38304 Wolfenbüttel, Germany

## Abstract

**Background:**

The identification of network motifs as statistically over-represented topological patterns has become one of the most promising topics in the analysis of complex networks. The main focus is commonly made on how they operate by means of their internal organization. Yet, their contribution to a network's global architecture is poorly understood. However, this requires switching from the abstract view of a topological pattern to the level of its instances. Here, we show how a recently proposed metric, the pairwise disconnectivity index, can be adapted to survey if and which kind of topological patterns and their instances are most important for sustaining the connectivity within a network.

**Results:**

The pairwise disconnectivity index of a pattern instance quantifies the dependency of the pairwise connections between vertices in a network on the presence of this pattern instance. Thereby, it particularly considers how the coherence between the unique constituents of a pattern instance relates to the rest of a network. We have applied the method exemplarily to the analysis of 3-vertex topological pattern instances in the transcription networks of a bacteria (*E. coli*), a unicellular eukaryote (*S. cerevisiae*) and higher eukaryotes (human, mouse, rat). We found that in these networks only very few pattern instances break lots of the pairwise connections between vertices upon the removal of an instance. Among them network motifs do not prevail. Rather, those patterns that are shared by the three networks exhibit a conspicuously enhanced pairwise disconnectivity index. Additionally, these are often located in close vicinity to each other or are even overlapping, since only a small number of genes are repeatedly present in most of them. Moreover, evidence has gathered that the importance of these pattern instances is due to synergistic rather than merely additive effects between their constituents.

**Conclusion:**

A new method has been proposed that enables to evaluate the topological significance of various connected patterns in a regulatory network. Applying this method onto transcriptional networks of three largely distinct organisms we could prove that it is highly suitable to identify most important pattern instances, but that neither motifs nor any pattern in general appear to play a particularly important role *per se*. From the results obtained so far, we conclude that the pairwise disconnectivity index will most likely prove useful as well in identifying other (higher-order) pattern instances in transcriptional and other networks.

## Background

Network analysis is increasingly recognized as a powerful approach to understand the organization of intracellular systems. The topology (i.e., the architecture) of a network describes how its elements are interconnected to one another, thereby providing the necessary structural basis for the subsequent analysis of the dynamics of the system. Various biological networks, such as metabolic or protein interaction networks, share global statistical features, i.e., (*i*) the small-world property referring to the shortest paths between any two vertices and highly clustered connections and (*ii*) the scale-free property, indicating that the vertex degrees follow a power-law distribution [1-7]. This implies a certain hierarchy of connectedness, as most vertices have a low degree and few vertices (hubs) have a markedly increased number of immediate neighbors.

This hierarchy is reflected in the modular organization of biological regulatory systems with each module performing its special functional task, separable from the functions of other modules [8,9]. Such a modularity of networks can be characterized topologically whereby their scale-free organization coincides with hierarchical modularity [3]. These hierarchical networks comprise many small clusters that are densely interconnected rather than consisting of independent groups of vertices [10]. Accordingly, modules may overlap with each other so that a nested type of organization is possible with smaller modules being part of bigger ones. It has been observed for various biological networks that the clustering coefficient of the vertices is approximately inversely proportional to their degree, which has been understood as the most important indication of hierarchical modularity of a network [3,11-13]. Understanding the organization of modules and their structural and functional roles emerges as a new challenge when studying biological networks. The corresponding analyses require proceeding from the level of vertices and their edges to the level of groups of these elements. It has been shown that 'network motifs' are an important feature of biological networks and may represent the simplest building blocks from which the bigger functional modules and whole networks are made [8,14,15]. They appear to relate to the lowest level of a hierarchical modularity.

Network motifs depict distinct topological patterns that occur more often in a given network than in random networks with the same size and degree distribution [14,15]. In contrast, significantly underrepresented patterns are known as anti-motifs [16]. Proteins belonging to specific motifs in the yeast protein interaction network tend to be highly conserved across species during evolution thereby underpinning that also their respective motifs may have an important, evolutionarily selected biological function [17,18]. The same network motifs have been found in diverse organisms from bacteria and yeast to plants and animals reviewed in [20]. The concept of network motifs as *the building blocks of evolution *has become one of the central topics in the analysis of complex networks. Usually, studies focus on how each network motif can carry out particular information-processing functions by means of its specific internal organization [19-23].

So far little attention has been paid to the role of motifs within a whole network, i.e., how they are embedded and how important they are for supporting the global architecture. Motifs are not isolated entities, but they are integral parts of the whole network. Thus, the targeted removal of the links among the vertices of all feed-forward loops and bi-fan clusters from the transcription regulatory network of *E. coli *fragmented this network into many small, isolated subgraphs [18]. Although this observation already indicates that motifs may be of big importance for the structure of a whole network it hides the impact of a single feed-forward loop or bi-fan representative in *E. coli*. It is unclear whether such a fragmentation is caused by a limited number of these representatives only and if the significance of a representative goes along with a particular kind of motif like the feed-forward loop. Furthermore, networks contain other topological patterns than motifs and it remains to be seen whether they take a minor role for the topology of a network [19]. Therefore, further studies are necessary and they require switching from the abstract view of a topological pattern to the level of their various representatives, the *instances of a pattern*. In general, a topological pattern depicts a unique kind of organization between a defined number of vertices which is given by the edges between these vertices. A pattern *instance *refers to a distinct set of vertices and all edges between them so that the arrangement of the edges reflects the respective pattern. To estimate the significance of such an instance for the topology of a whole network one has to consider how it relates to the rest of the network, i.e., its environment, and therewith which kind of influence it may have. No practical methods and theoretical approaches are yet available for this purpose.

To evaluate the topological significance of individual components in complex biological systems, we have recently introduced a new topological parameter – the pairwise disconnectivity index of a network's element [24]. Such an element might be a vertex (i.e., molecule, gene), an edge (i.e., reaction, interaction), as well as a group of vertices and/or edges. The pairwise disconnectivity index quantifies how essential an element is for sustaining the communication ability between all connected ordered pairs of vertices in a network. It can be viewed as a measure of sensitivity (robustness) of this network to the presence (absence) of each element. Here, we show how this concept can be used to estimate the topological significance of a pattern instance and to find out the role of the corresponding pattern within a whole network. Subsequently, we apply this approach exemplarily to the analysis of 3-vertex topological patterns in transcription networks from different organisms: a bacterium (*E. coli*), a unicellular eukaryote (*S. cerevisiae*) and higher eukaryotes (mammals, mainly human, mouse, and rat).

## Results

### The topological significance of a pattern instance

**Figure 1:**
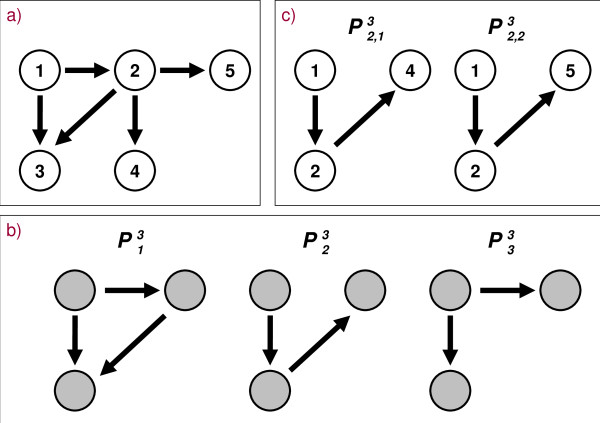
**Topological patterns and their instances in a network**. **A**: A toy network with the set of vertices *V *= {1,..., 5}. **B**: The entirety of all 3-vertex patterns  in the toy network. **C**: The two instances of the pattern .

Let *G *= (*V*, *E*) be a directed graph without multiple edges that represents a regulatory network, where the vertices *v *∈ *V *denote biological entities, e.g., proteins, genes or small molecules. Causal relationships between these entities are made up of directed edges *e *∈ *E*. A topological pattern is given by *n *connected vertices and the way they are connected with each other. The particular coherence which is described by a pattern is always based on all edges that exist between *n *vertices. The entirety of all distinct *n*-vertex patterns in *G *is then given by  where  is the *i*-th pattern consisting of *n *vertices. Actually, each pattern  represents a set of isomorphic connected subgraphs which have the same structural properties and differ only in the participating vertices. Accordingly, a pattern  comprehends a set of instances, i.e., , and each instance is a unique subgraph  of *G*, with the subset of vertices  ⊆ *V *and the subset of edges  ⊆ *E *(Figure 1). The edges in  are only incident to vertices in  and we denote them as the *intrinsic *edges of the pattern instance . Other edges, *e *∈ *E*\, do not contribute to the coherence of the vertices . Moreover, these *extrinsic *edges are part of the environment of  which describes how the pattern instance is embedded into the network. If there are more pattern instances  in *G *than in similar random networks, then the respective pattern  is called a motif. Consequently, the entirety of *n*-vertex patterns in *G *may contain several *n*-vertex motifs . Then, the motif  compasses its own representatives, the instances of the motif .

Following the logic of [24], we denote the *topological significance of a pattern instance * as how essential for all connections within a network it is. To quantify this significance we eliminate all edges of a pattern instance (i.e., its intrinsic edges ) and measure how this affects the number of connected ordered pairs of vertices in the network. An ordered pair of vertices (*i*, *j*)|i ≠ j and i, j ∈ V, is connected iff there is at least one path from vertex *i *to vertex *j *in *G*. Note, that the ordered pair (*i*, *j*) is different from (*j*, *i*) in a directed network. The more ordered pairs become disconnected upon the removal of all edges of a pattern instance, the higher is the topological significance of this instance for the whole network. We define the *pairwise disconnectivity index of a pattern instance*, *Dis*(), as the fraction of those initially connected pairs of vertices in a network which become disconnected if the intrinsic edges of the pattern instance  are removed from the network

(1)

In Eq. 1 *N *is the total number of ordered pairs of vertices in a graph *G *= (*V*, *E*) that are connected by at least one directed path of any length. It is supposed that *N *> 0, i.e., there exists at least one edge in the network that links two different vertices. *N' *is the number of ordered pairs of vertices in the subgraph *G' *= (*V*, *E'*) of *G *where *E*' = *E*/. Therefore, *G' *is the subgraph of *G *that results from removing the intrinsic edges of the pattern instance  from *G*. The pairwise disconnectivity index of a pattern instance ranges between 0 and 1, whereas zero indicates that the removal of its intrinsic edges does not disconnect vertices within the network and one denotes the cases when no pair of vertices is connected any more.

**Figure 2:**
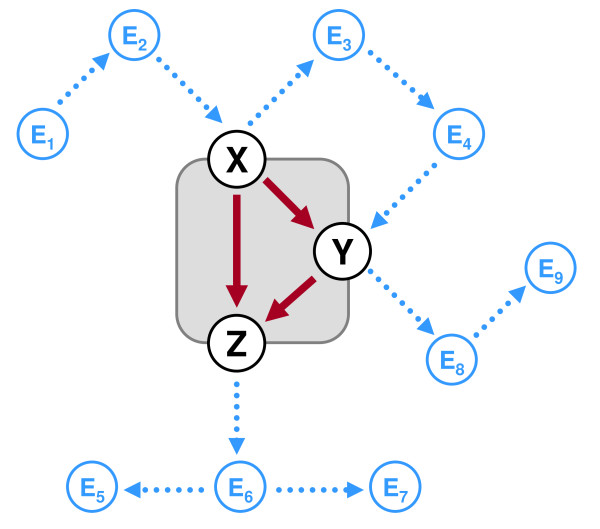
**The embedding of a feed-forward loop (FFL) instance into a network**. The FFL pattern is given here by the coherence between the vertices *X*, *Y*, *Z*. Therewith its only instance consists of these vertices and the intrinsic edges *X *→ *Y*, *Y *→ *Z*, *X *→ *Z*. How the FFL instance relates to the rest of the network is determined by those kinds of extrinsic edges that are attached to the vertices *X*, *Y*, *Z *(blue dotted edges). Other extrinsic edges link further vertices (blue vertices) in the environment of the FFL instances. The connection between a pair of vertices can be affected only then by the FFL instance if the paths linking them contain at least one of the intrinsic edges. For example, the connection between the pair (*E*_2_, *E*_6_) depends of the relation between the vertices *X*, *Y*, *Z*. In contrast, there still is an alternative path between the vertices *E*_2 _and *Y *that remains untouched. Note that the 'feed-forwarding' action of the FFL instance does not apply to those paths which cross only one intrinsic edge of this instance – e.g., path {*E*_1_, *E*_2_, *X*, *Y*, *E*_8_, *E*_9_} and path {*E*_3_, *E*_4_, *Y*, *Z*, *E*_6_, *E*_7_}.

Figure 2 illustrates how an instance of the feed-forward loop (FFL), one of the best studied network motifs [14,15,20-23], may affect the existing communication in a network. The FFL is a three-vertex pattern that is given here by the intrinsic edges *X *→ *Y*, *Y *→ *Z *and *X *→ *Z*. It is linked to the rest of a network by its vertices *X*, *Y*, *Z *where each of these can be at the start or end of an extrinsic edge (blue dotted edges). Further extrinsic edges are between other pairs of vertices in the environment of a FFL instance, e.g. the ordered pair (*E*_1_, *E*_2_). Whether a FFL instance can have an impact on the connection between two vertices depends on the kind of constituents of the paths that link them. If these paths consist of extrinsic edges only then the connection will not be affected upon the removal of the FFL (e.g., the pair (*E*_1_, *E*_3_)). Essentially, the FFL instance may be critical for those paths which include at least one intrinsic edge of the instance. However, that depends on the presence of alternative (i.e., parallel) paths between the corresponding vertices that use extrinsic edges only. For example, the pair (*E*_2_, *Y*) does not critically depend on the FFL instance due to the presence of another path (*E*_2_, *X*, *E*_3_, *E*_4_, *Y*) that includes no intrinsic edge of the instance. In contrast, the pair (*E*_2_, *E*_6_) looses its connection upon the deletion of the FFL instance though three parallel paths are connecting these vertices.

Usually, several instances of a particular pattern can be found in a network. For estimating the topological significance of the pattern itself the impact of its representatives has to be considered. We find that the average pairwise disconnectivity index of all instances of a pattern reflects this appropriately and define

(2)

as the *pairwise disconnectivity index of a pattern * that consists of *J *instances. With it Eq. 2 also states the topological significance of a randomly chosen instance of the pattern .

### Applying the pairwise disconnectivity index to the analysis of topological patterns in regulatory networks

We have applied our approach to the characterization of three-vertex topological patterns in transcription regulation networks from three different organisms: a bacteria (*Escherichia coli*) [14], a unicellular eukaryote (the yeast *Saccharomyces cerevisiae*) [15] and higher eukaryotes (mammals: human, mouse, rat) [25,26]. 3-vertex motifs were identified by means of the Z-Score as proposed by Alon and colleagues [15]. This normalized value states whether the abundance of a pattern in the real network exceeds its occurrence in a number of random ensembles: that is, a positive Z-Score refers to an over-representation in the real network, whereas a negative Z-Score means under-representation. Since there is no commonly accepted threshold Z-Score value for defining motifs, we consider patterns with Z-Score > 0 as motifs and all other ones as non-motifs. For the networks of *E. coli *and *S. cerevisiae *3-vertex motifs were already identified [14,15], whereas for the mammalian transcription network this is reported for the first time. To distinguish between different motifs many of which have no commonly accepted names, we used the identification numbers (IDs) of small connected graphs as it is provided by the FANMOD software [27,28]. The name of a pattern instance was generated by combining a prefix *E*, *Y *or *M *for referring to *E. coli*, *S. cerevisiae *or mammalian, respectively, with the corresponding ID followed by the pairwise disconnectivity index rank of the instance among all instances of a given pattern.

#### Bacterial transcription network

**Figure 3:**
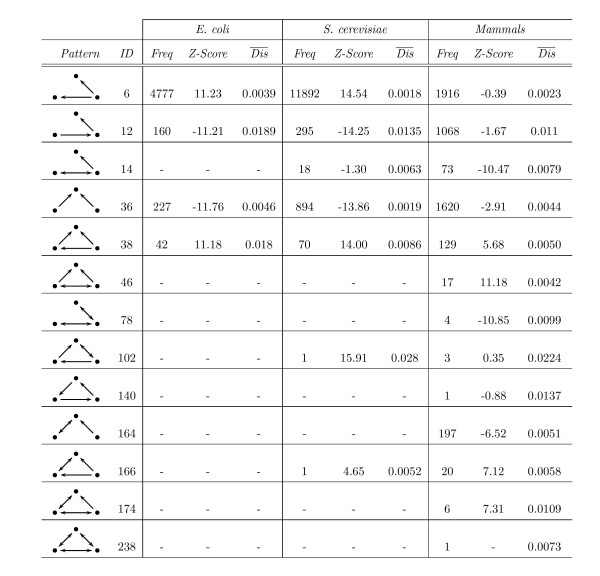
**3-vertex patterns in the transcriptional networks of *E. coli*, *S. cerevisiae *and *Mammals***. The column *Pattern *outlines the respective pattern. Its name can be found in the column *ID*. The column *Freq *denotes the number of occurrences of a pattern (a positive *Z-Score *indicates over-representation).  stands for the mean pairwise disconnectivity index of all instances of a pattern.

The *E. coli *transcription network consists of 418 vertices and 519 edges. It exhibits four 3-vertex patterns, two of which are motifs according to the Z-score criteria (Figure 3). One of these motifs (ID = 6) appears most frequently and seems to be part of larger motifs known as the single-input module [20]. The mean pairwise disconnectivity index of its instances is 0.0039: that is only about 0.4% of all connected pairs of genes become suspended when a randomly selected instance of this motif is deleted from the network. The second motif, ID = 38, is known as the feed-forward loop [14,15] and appears in the *E. coli *network less often than the previous, but its instances exhibit a higher average pairwise disconnectivity index (0.018). The patterns ID = 12 and ID = 36 are not over-represented here (negative Z-Score) and are therefore not ranked as motifs. The pattern ID = 12 denotes a chain-like structure where a gene regulates another one which itself regulates a third one. It is attributed to a pairwise disconnectivity index that ranges within the same scale as the feed-forward loop on average. In contrast, the pattern ID = 36, that abstracts the influence of two genes on a third one, has a much lower mean pairwise disconnectivity index than that of the ID = 12 pattern, but higher than that of the ID = 6 motif.

**Figure 4:**
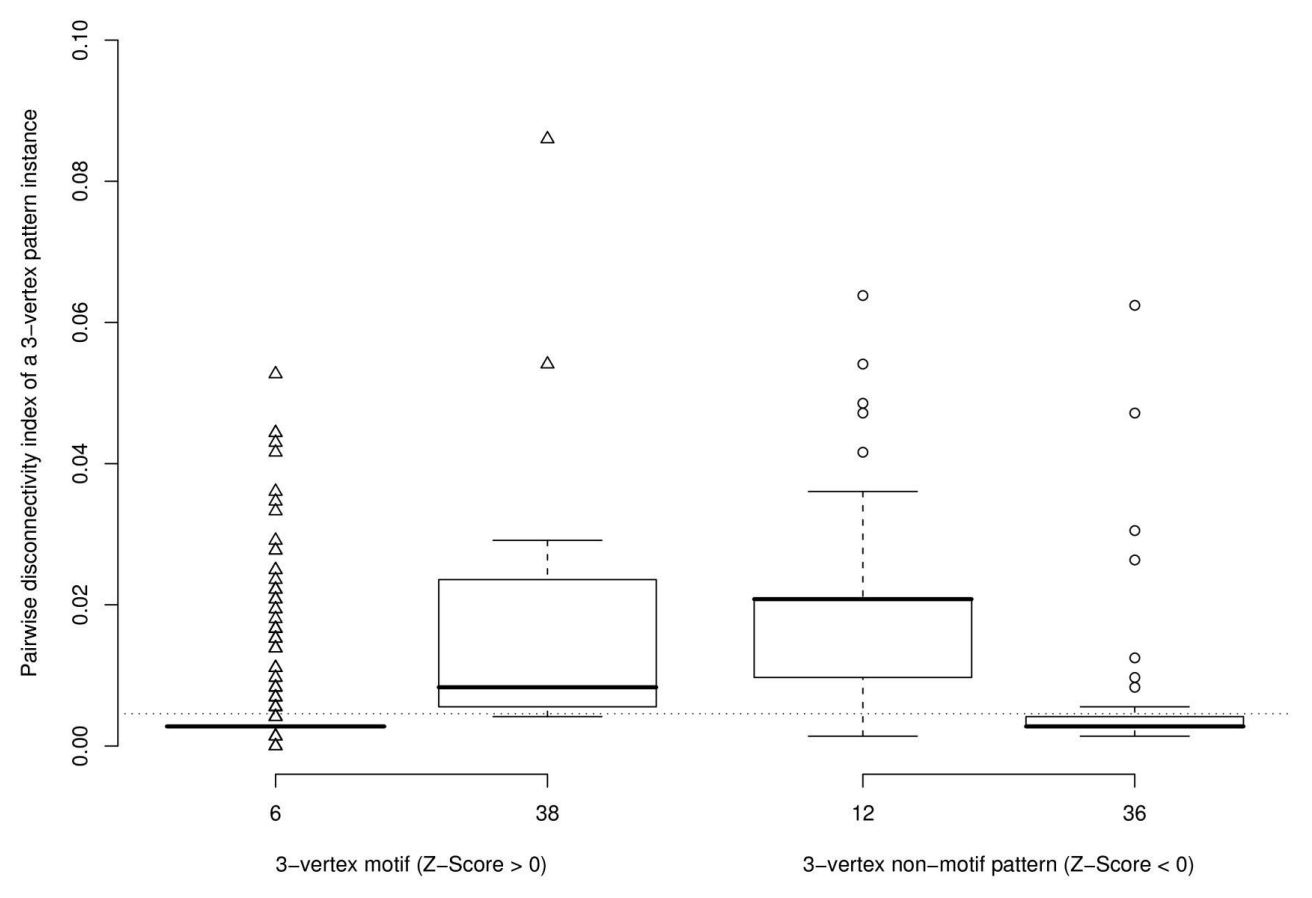
**The topological significance of 3-vertex pattern instances in *E. coli***. The left boxplot denotes the two 3-vertex motifs found in the network on the x-axis and the distribution of the pairwise disconnectivity index of their instances on the y-axis. The right boxplot constitutes this for patterns that are not over-represented in *E. coli*. The dotted line indicates the average pairwise disconnectivity index of all pattern instances in the network (0.0046). Note that one point may stand for several pattern instances.

The boxplots in Figure 4 show how the pairwise disconnectivity index is distributed among the instances of different 3-vertex patterns (see Figure 3, *E. coli*). The population of each pattern is very heterogeneous. Most instances exhibit a low pairwise disconnectivity index value. However, very few pattern instances cause a significant effect when deleted, thereby indicating that the network is vulnerable against a targeted removal of particular instances. While about 3% of all motif instances are not crucial for sustaining the connection between any gene pair, nearly 9% of them disconnect at least 1% of the gene pairs in *E. coli*. In contrast, the instances of non-over-represented patterns always disconnect at least one gene pair and one third of them 1% or more. In general, comparing the medians of pattern instances (shown in Figure 4 as a solid horizontal bar) indicates that motifs are not topologically more significant than the non-motif patterns.

**Figure 5:**
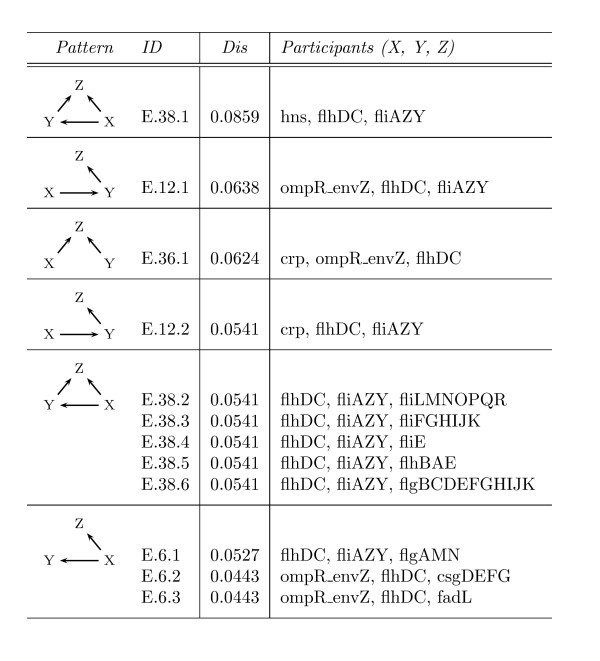
**The highest topologically significant 3-vertex pattern instances in *E. coli***. The column *Pattern *outlines the respective pattern of an instance. Its name can be found in the column *ID*. The column *Dis *refers to the pairwise disconnectivity index of the pattern instance. The column *Participants *denotes the set of genes involved in the instance and their locations within the pattern.

Nevertheless, the instance with the highest pairwise disconnectivity index in the *E. coli *network is a motif instance. This feed-forward loop consists of the genes *hns, flhDC *and *fliAZY *(Figure 5, ID = E.38.1). Interestingly, the gene *flhDC *is part of all pattern instances with a high topological significance, either together with the gene *fliAZY *or *ompR_envZ *(Figure 5). Like *hns *and *fliAZY*, the gene *flhDC *is involved in the synthesis of flagella in *E. coli*. A reduced activity of *flhDC *and *fliAZY *results in the loss of motility in *E. coli *[29,30] which has vital consequences for the bacteria. This can be the case for a loss of the *ompR_envZ *regulatory system too, which is known to play a critical role in stress response by regulating the transcription of porin genes in response to medium osmolarity [31]. Altogether, the high topological significance of the pattern instances in Figure 5 seems to reflect the importance of the few recurring interactions between these essential genes for *E. coli *adequately.

#### Yeast transcription network

The transcription network of *S. cerevisiae *consists of 688 vertices and 1079 edges. It features three additional patterns besides those ones that have already been identified in *E. coli*. A positive Z-Score is attributed to four patterns in *S. cerevisiae*, although the patterns ID = 102 and ID = 166 occur only once (Figure 3). Likewise to the observations from *E. coli*, the average topological significance of the motif ID = 6 is lower than that of the feed-forward loop. On average, a randomly selected FFL instance breaks the connection between less than 1% of all connected pairs of genes, which is lower than for instances of the pattern ID = 12. Their mean pairwise disconnectivity index is about 0.0135 and appears to be the highest of all patterns in the *S. cerevisiae *network with a negative Z-Score.

**Figure 6:**
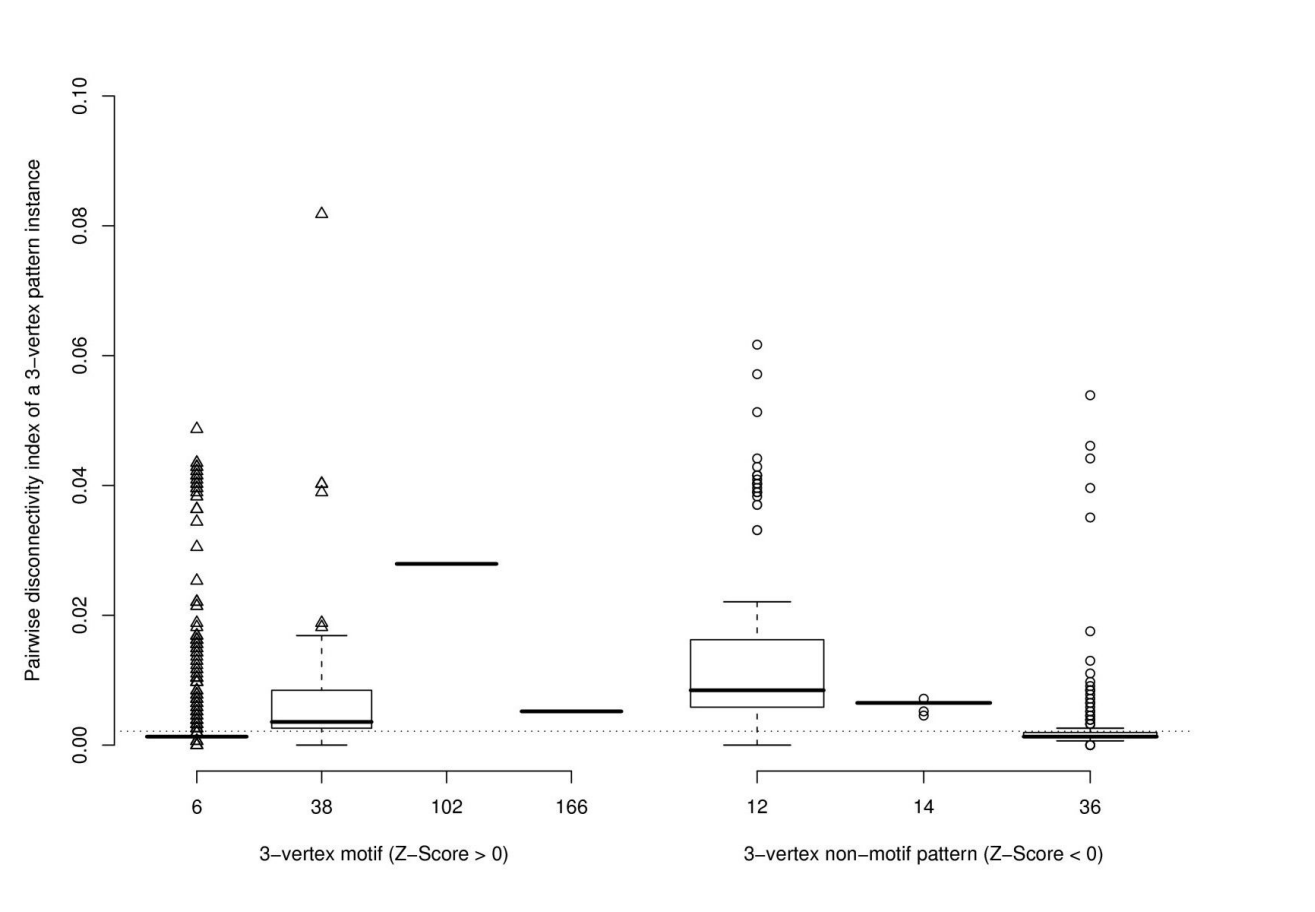
**Distribution of the pairwise disconnectivity index in 3-vertex patterns in *S. cerevisiae***. The left boxplot shows how the pairwise disconnectivity index of a motif instance (x-axis) is distributed in within the respective motif (y-axis). The boxplot on the right present a similar comparison for non-motif patterns in *S. cerevisiae*. The overall mean pairwise disconnectivity index of all pattern instances in the network (0.0021) is represented by the dotted line. One point may represent several pattern instances.

**Figure 7:**
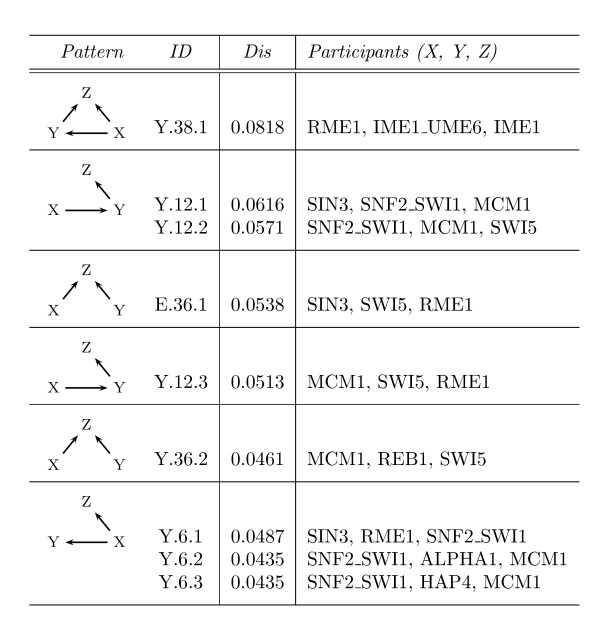
**The highest topologically significant 3-vertex pattern instances in *S. cerevisiae***. The column *Pattern *outlines the respective pattern of an instance. Its name can be found in the column *ID*. The column *Dis *refers to the pairwise disconnectivity index of the considered instance. The column *Participants *denotes the set of genes involved in the instance and their locations within the pattern.

Except for the pattern ID = 14, the pairwise disconnectivity index varies considerably for the instances of a pattern in this network (Figure 6). The respective patterns of the candidates with a high topological significance display positive Z-Scores as well as negative Z-Scores, which refer to over-representation and under-representation, respectively. Hence, motifs are not in favour for sustaining the pairwise connections between genes compared with non-motif patterns. In contrast to the *E. coli *network, the *S. cerevisiae *network seems to be more robust upon the elimination of a pattern instance, since much less of them have a notable effect on the existing pairwise connections between genes at all: The average pairwise disconnectivity index of a pattern instance is with 0.002 just half as high as in the *E. coli *network. Therewith, more alternative paths are at hand that strengthen pairwise connections between genes here so that also fewer instances cause a significant perturbation in the network (about 3% with *Dis *() ≥ 0.05 in yeast contrary to 10% in the *E. coli *network). Certainly, the overall impact of these pattern instances is comparable to the *E. coli *network (see Figures 5 and 7). A reason for this might be that such pattern instances are embedded in an alike fashion in both networks and may so have a similar influence on the existing connections.

The highest pairwise disconnectivity index is about 0.08 (Figure 6) and refers to a feed-forward loop instance that embodies the genes *RME1*, *IME1 *and *IME1_UME6 *(Figure 7). *RME1 *is known to encode a zinc finger protein that can repress the transcription of *IME1 *[32]. *RME1 *and *IME1 *are the master regulators of meiosis in *S. cerevisiae *[33-35]. An *ime1 *disruption prevents expression of almost all meiotic genes and all tested meiotic events [33]. *RME1 *is essential for sustaining the communication abilities between lots of gene pairs, similar to the genes *MCM1, SNF2_SWI1 *and *SWI5*. Gene *MCM1 *is central to the transcription control of cell-type specific genes and the pheromone response. The *SNF2/SWI *complex is an evolutionarily conserved ATP-dependent chromatin remodeling complex that plays an important role in DNA damage repair, DNA replication and stress response [36]. *SWI5 *activates the expression of cell cycle genes [37]. Altogether, these genes exert vital functions in *S. cerevisiae *and each of them appears quite frequently among the pattern instances with the highest topological significance.

#### Mammalian transcription network

The third network represents genes coding for transcription factors in mammalian species (human, mouse, and rat) and their interplay. This mammalian network consists of 279 vertices and 657 edges and has been extracted from the contents of the TRANSPATH^® ^database on signal transduction [25] and the TRANSFAC^® ^database on eukaryotic *cis*-acting regulatory DNA elements and *trans*-acting factors [26]. Unlike the other two networks it contains all of the thirteen possible 3-vertex patterns. Although five patterns display positive Z-Scores, only four of them indicate a clear over-representation (Figure 3). In addition, one might find it difficult to classify the pattern ID = 102 as a motif due to its low frequency. Nevertheless, the FFL is a motif in mammals and the only pattern that is over-represented in all three networks. Although its occurrence rises with the increasing density and complexity of the networks, its topological significance is decreasing notably. Actually, a low average pairwise disconnectivity index can be observed for almost all motifs in mammals, with motif ID = 174 as the only exception.

Three of the seven patterns with a negative Z-Score have been found in the networks of *E. coli *and *S. cerevisiae *too, but unlike in mammals the pattern ID = 6 is a motif in them. Yet, its average topological significance for these networks does not differ greatly. Similar applies to the pattern ID = 12 that exhibits one of the highest mean pairwise disconnectivity indices here as well. In contrast, just a minor role seems to be adopted by the pattern ID = 36 though it is the second most common one. Other non-motif patterns in the mammalian network are crucial for linking only 1% of gene pairs mostly on average. Nevertheless, their appearance is a hint on the more complex organization of transcription regulation in higher organisms. Thus, it seems to be convenient that the pattern ID = 238 can be found only here (Figure 3): it represents the mutual transcription control of three retinoic acid receptor isoforms with the vertices *RAR-alpha*, *RAR-beta *and *RAR-gamma*. Note that this pattern does not even occur in any random network of similar size and degree distribution. On the other hand, it is still surprising that the pattern ID = 164 appears nearly 200 times in the mammalian network, but neither in the network of *E. coli *nor in the network of *S. cerevisiae*.

**Figure 8:**
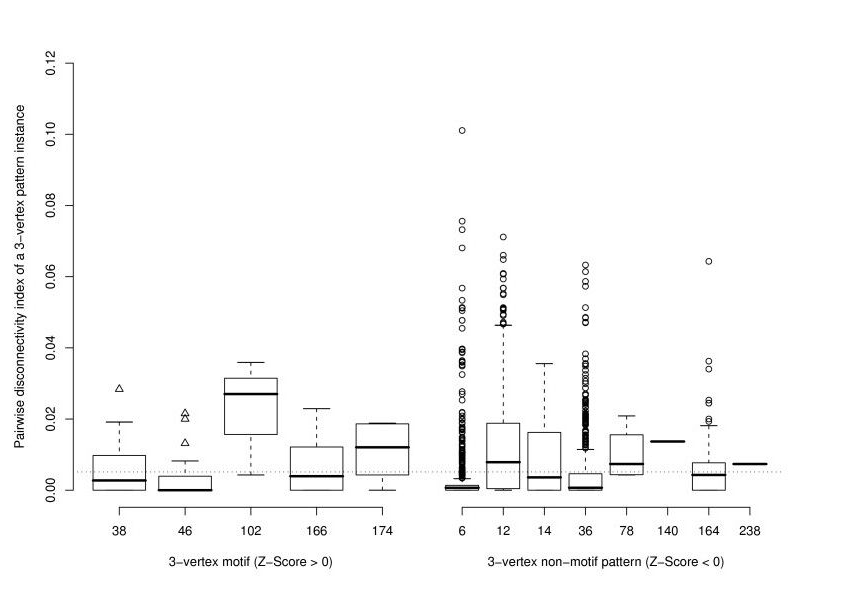
**The pairwise disconnectivity index of 3-vertex pattern instances in *Mammals***. The boxplot on the left indicates the distribution of the pairwise disconnectivity index in the 3-vertex motifs in the network. The same relation is pictured in the right boxplot for patterns with a negative or no Z-Score at all. The dotted line describes the mean pairwise disconnectivity index of all 3-vertex pattern instances in the network (0.0051). One point may represent several pattern instances.

Despite the overall low mean topological significance of the various patterns in the mammalian network, the pairwise disconnectivity index of their instances covers a broad range of values (Figure 8). This spreading is even stronger for non over-represented patterns and more noticeable as in the other two networks. Thus, a high topological significance does not go along with motifs here as well. However, this network is different with regard to the robustness of its architecture: About one third of all pattern instances do not affect any of pairwise connections between genes and more than 15% disconnect at least 1% of the gene pairs. No motif instance exhibits a pairwise disconnectivity index higher than 0.04. This can be found for non-over-represented patterns exclusively (ID = 6, 12, 36, 164).

**Figure 9:**
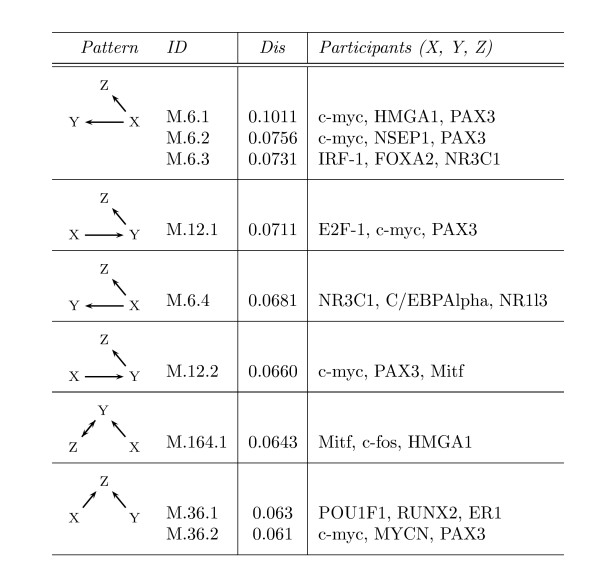
**The highest topologically significant 3-vertex pattern instances in *Mammals***. The column *Pattern *outlines the respective pattern of an instance. Its name can be found in the column *ID*. The column *Dis *refers to the pairwise disconnectivity index of the considered instance. The column *Participants *denotes the set of genes involved in the instance and their locations within the pattern.

The most intense perturbation outranks the topologically most significant pattern instances in the other two networks. Deleting this pattern instance, which comprises the genes *c-myc, HMGA1 *and *PAX3*, suspends the connections between 10% of all genes in the mammalian network (M.6.1, Figure 9). The proto-oncogene *c-myc *is engaged in diverse processes ranging from cell proliferation to apoptosis [38] and its interaction with *PAX3 *repeatedly occurs in the pattern instances with the highest topological significance (Figure 9). Such a frequent appearance has been observed for some genes in the networks of *E. coli *and *S. cerevisiae *too. Furthermore, these genes have been found to exert vital functions in their organism. The same applies for *PAX3 *and *c-myc *in mammals: The paired box gene 3 activates developmental genes (e.g., *Mitf*) and just as *c-myc *the loss of *PAX3 *is lethal [39]. It is interesting to note that all transcription factors encoded by the genes constituting the interlinked patterns M.6.1, M.6.2, M.12.1, M.12.2 and M.164.1 (Figure 9) play pronounced roles in cell proliferation (*E2F-1*, *c-myc*, *c-fos*, *HMGA1*, and *NSEP1*) or are important developmental regulators (*PAX3*, *Mitf*).

### A note on the joint deletion of intrinsic edges

The unusually often appearance of the same links (i.e., intrinsic edges) between genes in the pattern instances with the highest pairwise disconnectivity indices in all three networks raises the question of their contribution to the estimated significance of these pattern instances. Probably, the removal of individual intrinsic edges may already destroy the connection between many gene pairs so that their simultaneous removal is not as crucial. Otherwise they may have a significant non-additive impact taken together. However, answering this requires knowing the effect of deleting a single interaction (i.e., edge) in a network which can be accomplished in a similar way as for a pattern instance. It has been introduced as the pairwise disconnectivity index of an edge in [24] and specifies the fraction of ordered pairs becoming disconnected due to the removal of an individual edge.

**Figure 10:**
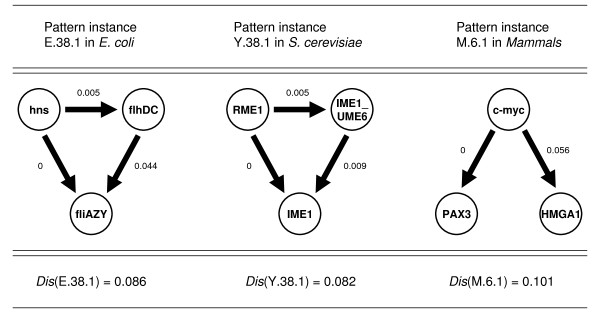
**The impact of the coherence between the genes in the pattern instances with the highest topological significance in the transcription networks**. Edge weights denote the impact of an individual edge for sustaining the pairwise connections between genes. In all three pattern instances, the intrinsic edges themselves fail to reproduce the impact as accomplished by their common elimination. The summarized effects of their separate removal are significantly lower as compared with the simultaneous deletion of the relation between the respective genes. Hence, these pattern instances affect only as whole entities those gene pairs that are linked by several alternative paths.

As a first attempt, this fraction has been estimated for each intrinsic edge of a pattern instance in the three networks and their sum has been opposed to the pairwise disconnectivity index of the respective pattern instance. Although such kind of comparison highlights just a tendency if and how far the intrinsic edges of a pattern instance act synergistically, it is already a way that works for all kinds of patterns independent of their specific arrangement. Figure 10 illustrates this approximation for the pattern instances with the highest pairwise disconnectivity index in each network. The edge weights denote the topological significance of an edge for the corresponding network, e.g., *Dis*(*hns *→ *flhDC*) = 0.005 for the edge from gene *hns *to *flhDC *in *E. coli*. Hence, the deletion of this interaction merely disconnects a half percent of all pairwise linked genes in *E. coli*. As expected, no effect is accomplished by removing the edge from *hns *to *fliAZY*, since there is always the alternative path via *flhDC*. In contrast, a relatively high pairwise disconnecivity index has been measured for the edge from *flhDC *to *fliAZY*. But still, the summarized effect of deleting these intrinsic edges separately from the *E. coli *network (0.049) is considerably lower as compared with the topological significance for the whole pattern instance, *Dis*(*E.38.1*) = 0.086. The same holds for the other two pattern instances in Figure 10 as well. Therewith a much stronger impact on pairwise connections between genes clearly exists due to the coherence of the intrinsic edges.

**Figure 11:**
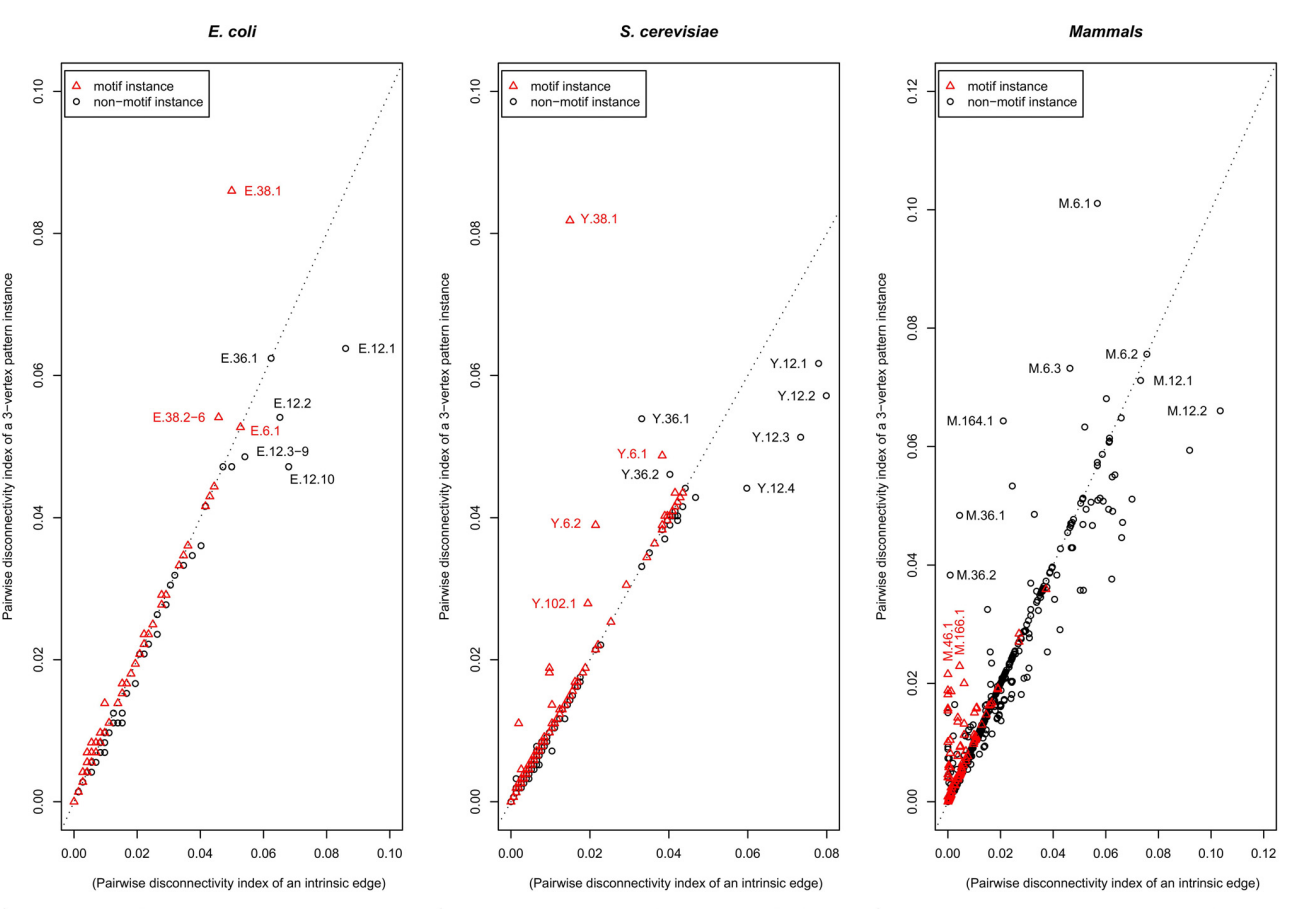
**The joint deletion of the intrinsic edges of a pattern instance may synergistically reduce connections between genes in the *E. coli*, *S. cerevisiae *and *Mammals *networks**. The pairwise disconnectivity index of a pattern instance (motifs are drawn as red triangles, others as black circles) is outlined on the y-axis. By contrast, the x-axis denotes the fraction of gene pairs becoming disconnected upon the deletion of a single intrinsic edge, summarized for all intrinsic edges of a given pattern instance. The diagonal dotted lines indicate the cases when the impact of the concurrent elimination of all intrinsic edges of a pattern instance does not differ from the sum of impacts provided by the separate removal of the same edges. Hence, pattern instances that are drawn below these lines include intrinsic edges that have an overlapping in their impacts: they can disconnect the same gene pairs. Finally, the position of a pattern instance above the dotted lines shows that some of its intrinsic edges are parts of alternative (i.e., parallel) paths between two genes. Such genes do not become disconnected when only one intrinsic edge is eliminated, but some of them do upon simultaneous removal of all intrinsic edges of the pattern instance: i.e., the joint removal exerts a higher than merely additive effect.

Whether this can be generalized for all pattern instances found in the three networks is shown in Figure 11. Most pattern instances in the three networks cluster near the diagonal since the joint removal of their intrinsic edges disconnects approximately the same number of gene pairs as the separate elimination of them does. However, some exceptions have been found, especially among those patterns that exhibit a high pairwise disconnectivity index *per se*.

A pattern instance is positioned below the diagonal dotted lines in Figure 11 due to considerable overlapping in the sets of pairwise linked genes which become disconnected upon the separate removal of the intrinsic edges of the instance. For example, consider how the vertices *1 *and *5 *in Figure 1A are linked. To disconnect them it is enough to delete one of the edges *1 *→ *2 *or *2 *→ *5 *at a time. Such kinds of dependencies seem to exist in larger scales in the analyzed networks pinpointing to lots of gene pairs that are connected in a linear chain-like manner as reflected by the pattern ID = 12 (Figure 3). There are almost no independent alternative paths between such gene pairs so that the connection between them is very sensitive upon the deletion of a single intrinsic edge. Therewith, the pattern ID = 12 is contained virtually exclusively amongst the pattern instances below the diagonal dotted lines in Figure 11.

The concurrent elimination of the intrinsic edges of a pattern instance located above the diagonal dotted lines breaks also pairwise connections between genes that are not so easily assailable as described above. At least two paths between such genes exist, each using a unique combination of intrinsic edges. Thus, they cannot be affected by eliminating a single intrinsic edge only. For example, in Figure 2 there are three paths linking vertex *E*_2 _with *E*_6_: The first one includes the intrinsic edge *X *→ *Z*. The second consists of the intrinsic edges *X *→ *Y *and *Y *→ *Z *whereas the third path contains only the edge *Y *→ *Z*. However, no matter which of the intrinsic edges is deleted, the vertex pair (*E*_2_, *E*_6_) remains untouched since at least one of the three paths is still present. Their connection is disrupted only if the whole pattern is deleted. Such dependencies can be observed in Figure 11 for few pattern instances in *E. coli*, but increasingly in the other two networks. This trend is most distinctive in the mammalian network. Besides the pattern instances with a high pairwise disconnectivity index, a considerable number of motif instances appear in the lower left corner of the plot for the mammalian network (Figure 11, red triangles): their intrinsic edges have an extremely small or even no impact at all on pairwise connections between genes. But as motif instances, they are a bottleneck for linking many gene pairs.

## Discussion

### A new method to asses the global role of patterns and motifs

The work presented here describes a method that has been proved to be suitable for evaluating the role of topological patterns within a network. This holds true regardless of the size and complexity of these patterns. The method assesses the significance of a pattern depending on the contribution of its instances, i.e. connected subgraphs, for the connectivity of a network. The approach is based on the technique described previously in [24], which estimates the necessity of a network element (e.g., a vertex or an edge) for sustaining the communication ability between connected pairs of vertices in a network. This is accomplished in a similar way as wet experiments in a lab: a gene (corresponding to a vertex in a graph) is knocked out and the effect of this removal is observed in the considered context. The same may be applied to a reaction (an edge in the graph), when a gene has been mutated and the encoded product (vertex) is still present, but unable to undergo a certain reaction.

In this work, we have proposed to proceed likewise for pattern instances, but disturbing the interactions between the involved vertices rather than eliminating the vertices themselves. Consequently, only the causal links between these vertices are destroyed and therewith the respective pattern is removed in a minimally invasive way. This is conducted without making any *a priori *assumptions on the analyzed network and its properties. In contrast to the attempt made in [18], we destroy the coherence between the edges of only one single pattern instance at a time, leaving the remainder of the network intact. On the one hand, different impacts on the network connectivity exerted by the various instances of a pattern can thus be discovered. On the other hand, the topological role of a pattern can be determined more realistically since an overrating is avoided.

### 3-Vertex patterns in transcriptional networks

We exemplarily applied the method developed and proposed here to the analysis of transcriptional regulation networks of three very distinct taxa (*E. coli, S. cerevisiae *and mammals, i. e. human, mouse, and rat); for simplicity, we focused here on 3-vertex topological patterns in these networks, but the method can easily be adopted to the analysis more complex and larger patterns. A first check of which of the thirteen possible 3-vertex patterns are present in these networks at all revealed that all of them can be found in the mammalian network, the *S. cerevisiae *network contains seven and that of *E. coli *only four of them. Moreover, these latter four patterns are shared by all three networks. Amongst them, only the "feed-forward loop" is statistically over-represented and, thus, could be considered as a "motif" (Figure 3).

As to be expected, the abundance of a pattern decreases with its complexity: Thus, 3-vertex patterns with two edges occur much more frequently than those with three edges, etc. The order of the abundance is almost the same in all three transcription networks. It is of interest that the network patterns "coupled feedback loop" (Figure 3, ID = 78) and "3-vertex-circuit" (Figure 3, ID = 140) do not exist in the networks of *E. coli *and *S. cerevisiae *and are clearly under-represented in the network of mammals (Figure 3), although they are widespread in signaling circuits of various bacterial and eukaryotic organisms [40-44]. We assume that this is an intrinsic property of transcriptional networks and cannot be explained by the incompleteness of the underlying knowledge, since other patterns of similar complexity (e.g., the mentioned feed-forward loop) are not consistently under-represented among these three networks.

All networks studied here appear to be rather robust against the elimination of a randomly chosen pattern instance. Therewith, the various 3-vertex patterns in these networks display a low topological significance on average. Mostly, the overall majority of the instances of a pattern have a rather small effect on the existing pairwise connections between genes, in most cases even less than 1% of all pairwise connections are affected.

### Motifs do not seem to be more important than non-motif patterns for the global architecture of a network

Also the motifs among the 3-vertex patterns examined did not exhibit a generally higher importance for the connectivity of the whole network than non-motif patterns, as one might have expected. This is, however, in agreement with previous studies on the evolutionary and functional assessment of motifs in the regulatory networks of different yeasts, which have provided evidence that motifs are not subject to any particular evolutionary pressure to preserve the corresponding interaction pattern [45,46]. No simple relationships have been found between evolutionary conservation and over-representation of network patterns, on the one hand, and their functional enrichment, on the other hand, in the yeast regulatory network [42]. In accordance with these observations, our results indicate that there is no positive correlation between the abundance (i.e., over-representation) of a network pattern and its topological significance. Thus, focusing on motifs exclusively rather than searching for important pattern instances in general would have lead to a completely different and deceptive picture.

### Pattern instances can be identified that are crucial for the connectivity of the network

In spite of the generally low impact of all types of patterns (including motifs) found in the analyzed networks, a few pattern instances cause a significant perturbation upon their removal. This trend is manifested in the heterogeneous distribution of the pairwise disconnectivity index among all the instances of a pattern (Figs. 3, 4, 5). Topologically, this may originate from the way how a pattern instance is embedded, i.e., its particular position within the whole context of the respective network. Biologically, such heterogeneity might be caused by the influence of the genes in the network that are forming a pattern instance. In the networks, the topologically most significant pattern instances consist preferably of genes that provide basic functions for the organism. Interestingly, most of these instances belong to one of the patterns that are shared by the three networks, which may emphasize the importance of these patterns. Furthermore, such instances may indicate locations within the networks rendering them vulnerable upon a targeted removal.

Among the pattern instances that are of particular importance for the network connectivity, motif instances again do not play a predominant role over instances from non-over-represented patterns. In the mammalian network, most of the outliers even belong to the non-motif patterns. Altogether, our data support the view that far not all instances of any pattern (motif or not), but only few of them may play specific functional roles [47] and thereby exhibit a strong impact on pairwise connections between genes in transcription networks.

### Pattern instances of high topological significance tend to form clusters

In all the networks analyzed here, a limited number of genes repeatedly appears in the pattern instances displaying the highest topological significance. For example, in *E. coli *the gene *flhDC *is part of all pattern instances that disconnect at least 4% of the gene pairs, preferably together with the genes *fliAZY *or *ompR_envZ*. Similar observations can be made in *S. cerevisiae *for the genes *MCM1, SIN3, SNF2_SWI1 *and *SWI5*. Likewise in the mammalian network, the interaction between the genes *c-myc *and *PAX3 *participates in many of the pattern instances with a high pairwise disconnectivity index. Altogether, the common occurrence of genes and interactions between them underlines the key importance of these constituents for the corresponding organism. All these genes are engaged in important processes and at least in *E. coli *and *S. cerevisiae *they are crucial for linking a significant number of gene pairs [24]. Hence, their damage can be lethal for the respective organism. Furthermore, these pattern instances are not located in different regions of a network. They are connected with each other and seem to form a bigger pattern cluster that controls a lot of pairwise connections between genes in these networks.

### Edges of pattern instances display synergistic effects

In many cases, the intrinsic edges of a pattern instance contribute to its pairwise disconnectivity index in a synergistic manner, i.e., the simultaneous removal of the respective edges exerts a much higher than merely additive effect (Figure 11). Although the approach we used for this purpose is a conservative approximation, it shows a principal tendency in these networks. More exact computations of this feature may be desirable but developing suitable algorithms for this, which have to take into account the particular characteristics of every pattern separately, was beyond the scope of this paper. However, we find that our approach was adequate to disclose clearly that the intrinsic edges of certain pattern instances display synergistic effects. This is the case for the pattern instances with the highest pairwise disconnectivity index in each of the three networks. Some other candidates have been found in *E. coli *and increasingly more in *S. cerevisiae *and mammals. This trend goes along exactly with the increasing density of the networks (1.2 edges per vertex in the *E. coli *network, 1.6 in *S. cerevisiae *and 2.3 in *Mammals*). The reason for this is on the hand: a more densely connected network provides a higher average vertex degree and thereby offers more alternative paths between pairs of vertices. These paths need not to share a similar set of edges, i.e., the connection of a pair is becoming more robust requiring more edges to be removed in order to disconnect it.

### Prospects of the proposed method

It should be noted that the observations reported here have been made for the networks as they are known at present. In particular the mammalian network may still suffer from incomplete knowledge. However, our method can be used for monitoring changes in such networks obtained from updated pathway databases like TRANSPATH^® ^[25] in the future. We see our results as the beginning of a large work which may consider the analysis of increasingly larger patterns including more than 3 vertices. More regulatory networks of various types (e.g., signal transduction networks, protein-protein interaction networks, gene expression networks) from different organism must be considered and tested in this regard in future as well. First attempts with signaling networks have confirmed the basic conclusions drawn here in spite of small characteristic differences in some details. Thus, we feel that the basic trends reported here will hold true for the more complete transcriptional as well as for other types of networks that will come up in future with increased reliability of high-throughput approaches and their systematic application.

On the other side, our method provides for the first time the possibility to assess the impact of patterns and motifs in general as well as individual pattern instances onto the overall connectivity of a graph. It is therefore suitable to identify bottlenecks in a biological network, which may be particularly important for the normal function of a cell, and may be top candidates to investigate disease mechanisms related to these functions. Since it identifies individual components in a network (vertices, edges, or pattern instances), it works independently of any *a priori *knowledge about the statistical over- or under-representation of certain network features. Though our approach was developed for the analysis of biological regulatory networks, it seems to be suitable for the analysis of other networks regardless of the particular nature of processes they represent (e.g., ecological, social, technical networks).

## Conclusion

We have developed a new method that quantifies how the elimination of a topological pattern instance affects the existing communication abilities within a network. We have applied this method exemplarily to the analysis of 3-vertex topological patterns and their instances in the transcription networks from a bacteria, yeast and mammals.

The elimination of most 3-vertex pattern instances does not drastically affect the global structure of transcription networks. However, these networks are vulnerable upon a targeted perturbation of few pattern instances. In these cases, the links between their genes contribute to the pairwise disconnectivity index of the pattern instance in a synergistic manner, i.e., the simultaneous removal of the respective edges exerts a much higher than merely additive effect. The topological significance of an instance does not easily correlate with the abundance of the respective pattern in a network. Although motifs might play an essential role in their respective local contexts, they do not seem to be more important than non-motif patterns for the global architecture of a network. Rather, the topological role of a pattern instance is unique and mainly determined by its location and the way how it is embedded in a given network.

## Methods

### Network databases

Literature-based databases of experimentally verified direct relationships for *Escherichia coli *[14] and *Saccharomyces cerevisiae *[15] have been used where *E. coli *V1.1 and *S. cerevisiae *V1.3 are available at . The mammalian network of transcription factor genes (human, mouse, rat) was retrieved from the TRANSPATH^® ^Professional database (release 8.3, made in 2007) on signal transduction [25] and TRANSFAC^® ^Professional database (release 11.3, made in 2007) on eukaryotic *cis*-acting regulatory DNA elements and *trans*-acting factors [26]. The network describes the causal relationships between genes that are coding for transcription factors, based on the regulation of these genes from transcription factors. However, the transcription factors themselves are not part of the network, i.e., the interaction chain "gene A codes for transcription factor A regulates gene B" has been summarized to: "gene A → gene B", which is a commonly used technique when inferring gene regulatory networks. Furthermore, genes are represented at the level of "ortholog abstraction", at which all species-specific data (human, mouse, rat) that refer to mammalian genes have been summarized to corresponding generic entries.

Selected genes (vertices) in the yeast and mammalian transcription networks were checked for their viability using the BIOBASE Knowledge Library™  and the *Saccharomyces *Genome Database (Stanford Genomic Resources [48]).

### Pattern analysis

The networks were scanned for 3-vertex topological patterns using the FANMOD software with default settings [27,28]. The statistical significance of the network motifs was evaluated by means of the Z-Score [15], *Z *= (*M*_*real *_- *M*_*rand*_)/*SD*, where *M*_*real *_and *M*_*rand *_are the numbers of appearance of the motif in the real network and the randomized networks, respectively. *SD *is the standard deviation. The sign of edges (such as 'positive' for activation or 'negative' for inhibition) is not considered.

The pairwise disconnectivity index was calculated using the DiVa software [49]. The statistical analysis was accomplished with R [50].

### The pairwise disconnectivity index of an edge

For estimating the impact of a single intrinsic edge on the existing pairwise connections between genes we have applied the pairwise disconnectivity index on an edge as defined in [24]. In this manner it states the fraction of those ordered pairs of vertices that have been disconnected upon the removal of an edge, i.e., . Similar to Eq. 1, *N *is the number of linked ordered pairs of vertices in a network and we assume *N *> 0. The term *N' *stands for the number of connected ordered pairs of vertices in the network we obtain when deleting the edge *e*. Hence, *Dis*(*e*) = 0 the edge *e *is not crucial for linking at least of vertex pair. In contrast, *Dis*(*e*) = 1 if no vertex pairs remains connected.

## Authors' contributions

APP and BG conceived the study, interpreted the data and drafted the manuscript. BG carried out the programming and performed the statistical analysis. EW participated in the coordination of the study and gave final approval of the version to be published. All authors read and approved the final manuscript.
